# The Association between Polymorphism of *INSR* and Polycystic Ovary Syndrome: A Meta-Analysis

**DOI:** 10.3390/ijms16022403

**Published:** 2015-01-22

**Authors:** Chun Feng, Ping-Ping Lv, Tian-Tian Yu, Min Jin, Jin-Ming Shen, Xue Wang, Feng Zhou, Shi-Wen Jiang

**Affiliations:** 1 Department of Reproductive Endocrinology, Women’s Hospital, School of Medicine, Zhejiang University, Hangzhou 310006, China; E-Mails: doctorfc@zju.edu.cn (C.F.); lvpingping@zju.edu.cn (P.-P.L.); 21318158@zju.edu.cn (T.-T.Y.); 20618235@163.com (F.Z.); 2 Department of Reproductive Endocrinology, the Second Affiliated Hospital of Zhejiang University School of Medicine, Hangzhou 310009, China; E-Mail: jinmin76@hotmail.com; 3 Department of Orthopedics, the First Affiliated Hospital of Zhejiang Chinese Medicine University, Hangzhou 310018, China; E-Mail: shenjinmg@gmail.com; 4 Department of Biomedical Sciences, School of Medicine, Mercer University, Savannah, GA 31404, USA; E-Mail: wang2499@umn.edu

**Keywords:** single nucleotide polymorphism (SNP), insulin receptor gene (*INSR*), polycystic ovary syndrome (PCOS), meta-analysis

## Abstract

Polycystic ovary syndrome (PCOS) is the most common gynecological endocrine disorder. The genetic background is believed to play a crucial role in the pathogenesis of PCOS. In recent years, the role of insulin receptor (*INSR*) polymorphisms in PCOS predisposition has attracted much attention. We performed a meta-analysis to investigate the association between the single nucleotide polymorphisms (SNPs) of *INSR* and PCOS. Published literature from Pubmed, Embase, and Cochrane CENTRAL was retrieved up until 7 August 2014. A total of 20 case-control studies including 23,845 controls and 17,460 PCOS cases with an average Newcastle-Ottawa quality assessment scale (NOS) score of 6.75 were analyzed. Ninety-eight SNPs distributed in 23 exons and the flanking regions of *INSR* were investigated, among which 17 SNPs were found to be associated with PCOS. Three SNPs detected in more than three studies were selected for further analyses. Twelve studies including 1158 controls and 1264 PCOS cases entered the analysis of rs1799817, but no significant association was found for every genotype (*p* > 0.05). Further subgroup stratification by ethnicity and weight did not lead to discovery of significant correlation (*p* > 0.05). For rs2059806, four studies including 442 controls and 524 PCOS cases were qualified for meta-analysis, and no significant association with PCOS was found for any genotype (*p* > 0.05). Four studies including 12,830 controls and 11,683 PCOS cases investigated the correlation between rs2059807 and PCOS, and five of the six cohorts indicated a significant impact. Our current meta-analysis suggests no significant correlation between rs1799817/rs2059806 SNPs and susceptibility of PCOS, while rs2059807 could be a promising candidate SNP that might be involved in the susceptibility of PCOS.

## 1. Introduction

Polycystic ovary syndrome (PCOS) is the most common gynecological endocrine disorder that is characterized by ovarian dysfunction, hyperandrogenism, and polycystic ovary morphology [1]. It is associated with increased risks of infertility, impaired glucose tolerance, type 2 diabetes mellitus, and metabolic syndrome [2]. While 6%–17% of reproductive-age women worldwide suffer PCOS [3,4], some ethnicities, such as South Asian, have higher incidence rates [5]. The exact molecular mechanism and cellular pathways underlying this disorder are still unclear. Etiological studies demonstrated the significance of genetic susceptibility for the pathogenesis of PCOS [6]. It is believed that multiple, instead of single genes, such as fibrillin-3 (*FBN3*), fat and obesity associated gene (*FTO*), insulin (*INS*), insulin receptor (*INSR*), insulin receptor substrate 1 (*ISR1*), DENN/MADD domain containing 1A (*DENND1A*), thyroid adenoma associated protein (*THADA*), and luteinizing hormone receptor (*LHR*), may contribute to the development of PCOS [7,8]. For example, *DENND1A* may alter the aminopeptidase activity of endoplasmic reticulum and is involved in the development of PCOS [9].

*INSR* gene is located at the short arm of chromosome 19 and comprises 22 exons. The *Insr* knockout mice manifested extreme insulin resistance [10]. Insulin resistance may up-regulate LH secretion in pituitary, testosterone production in theca cells, and P450scc activity in granulosa cells, which may disturb follicular maturation and lead to PCOS [7]. Considering that accumulated data showed a strong association between *INSR* and insulin resistance [11], allelic polymorphism of *INSR* may impose a genetic predisposition for the development of PCOS. Indeed, a series of studies have been conducted to investigate the relationship between single nucleotide polymorphisms (SNPs) of *INSR* and PCOS. However, data from these studies appeared to be highly controversial [12,13]. Although a meta-analysis published in 2010 has reviewed then available data, and found no association between the SNPs of *INSR* and PCOS [14], two Genome-Wide Association Studies in 2011 [15] and 2012 [16] pointed to a positive association between the polymorphisms of *INSR* and PCOS. These findings ignited a renewed interest in the topic. Since then more studies have been performed, and both positive and negative results have been reported.

As there have been plenty of studies exploring the role of *INSR* polymorphisms in PCOS, and the results often contradict each other, we carried out an updated meta-analysis covering recent data. The findings will provide useful information for elucidating the relationship between the polymorphisms of *INSR* and the risk of PCOS.

## 2. Results

### 2.1. Selection of the Studies

**Figure 1:**
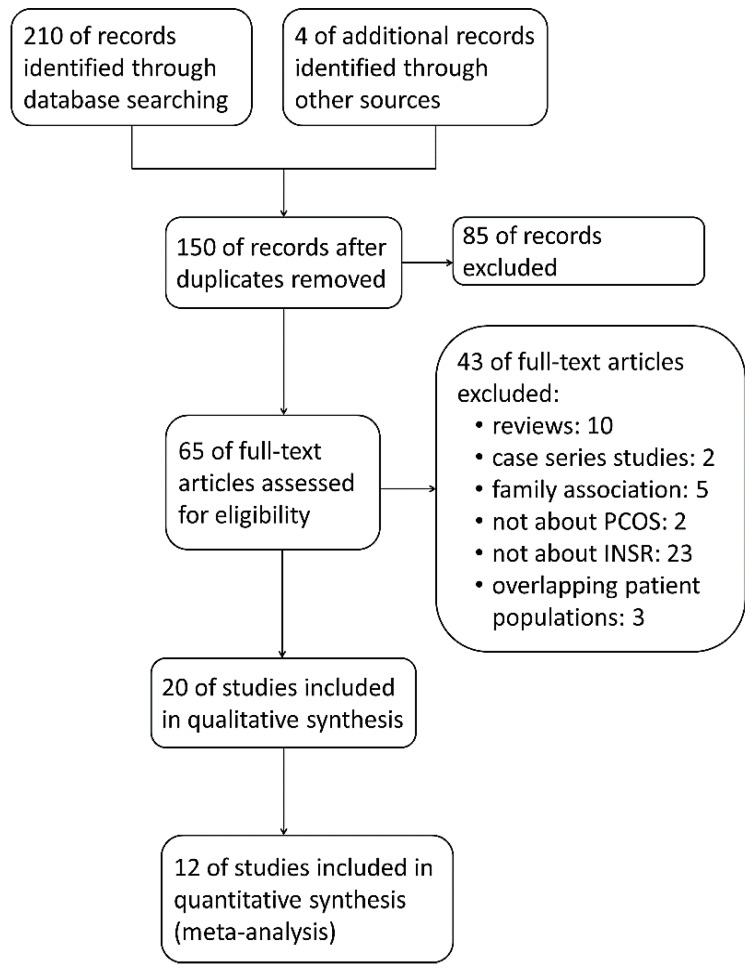
Flow chart of study selection for meta-analysis of insulin receptor (*INSR*) single nucleotide polymorphisms (SNPs) and risk of polycystic ovarian syndrome (PCOS).

A total of 150 articles were recognized by database searching and reference reading (Figure 1), among which 85 studies were excluded based on information from titles and abstracts. Due to the exclusion reasons listed in the flow chart, including overlapping patient populations in three articles [17,18,19], 20 studies remained for the qualitative synthesis. Eight studies were removed because the involved SNPs were detected in fewer than three studies [20,21,22,23] or the failure to obtain original genotype data [15,16,24,25]. 12 studies were finally selected for meta-analysis.

**Figure 2:**
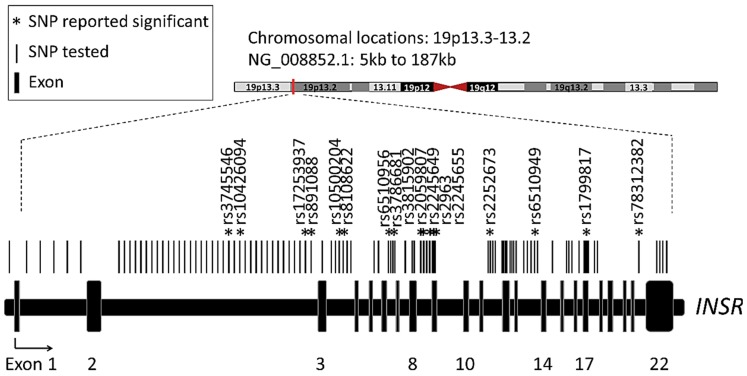
Map of investigated *INSR* SNPs in former studies. *: SNPs that were found to distribute differentially between PCOS and control.

The initially included studies consist of 10 from Asia, seven from Europe, two from North America, and one from South America. These studies cover totally 17,460 cases and 23,845 controls (Table 1). All of these studies were case-control studies published between 1994 and 2013. All were published in English except for one in Chinese. Since the ethnicities in some countries such as Brazil are complicated, it is difficult to define the ethnicities in different studies. As shown in Figure 2 and Table 2, a total of 98 different SNPs were investigated in these studies, which distributed in 23 different exons and the flanking regions of *INSR* gene. We picked the SNPs that were examined in more than three studies for further meta-analysis. The PCOS patients and controls were genotyped for rs1799817 SNP in 12 studies, for rs2059806 in four studies, and for rs2059807 in four studies.

**Table 1:** The characteristics of the studies included for qualitative analyses.

Study	Year	Country	Ethnicity	Diagnosis Criteria	Number		Methodology	NOS Score
					Control	PCOS		
Conway [26]	1994	UK	ND	ND	8	22	SSCP, sequencing	6
Talbot [27]	1996	UK	ND	Oligo/amenorrhea, hirsuitism, raised testosterone/androstenedione, polycystic ovaries	5	24	SSCP, sequencing	6
Siegel [12]	2002	USA	White	Oligomenorrhea, hyperandrogenism, polycystic ovaries	136	99	RFLP	7
Chen [28]	2004	China	Chinese	Oligo/amenorrhea, LH/FSH ≥ 2.5 or TT ≥ 1.56 nmol/L, polycystic ovaries, without endocrine disease or hypertension	40	120	RFLP	8
Jin [20]	2006	China	Chinese	Rotterdam criteria 2003	107	109	SSCP, sequencing	6
Lee [29]	2008	Korea	Korean	Rotterdam criteria 2003	100	134	sequencing	7
Mukherjee [30]	2009	India	Indian	Rotterdam criteria 2003	144	180	sequencing	7
Unsal [13]	2009	Turkey	Caucasian	Rotterdam criteria 2003	50	44	RFLP, sequencing	8
Hanzu [21]	2010	Romania	ND	Rotterdam criteria 2003	111	115	sequencing	7
Goodarzi [22]	2011	USA	ND	Discover cohort: NIH criteria 1990; replication cohort: Rotterdam criteria 2003	3758	801	Taqman assay, GWAS	8
Chen [15]	2011	China	Chinese	Rotterdam criteria 2003	6687	4082	GWAS, LDR	7
Cirilo [31]	2012	Brazil	Brazian	Rotterdam criteria 2003	105	117	RFLP	6
Ranjzad [32]	2012	Iran	ND	NIH criteria 1990	181	181	RFLP, sequencing	8
Shi [16]	2012	China	Chinese	Rotterdam criteria 2003	9594	9736	GWAS, LDR	7
Kashima [33]	2013	Japan	Japanese	Diagnostic criteria of the Japan Society of OBGY 2007	99	61	Taqman assay	7
Skrgatic [34]	2013	Croatia	Crotian	Rotterdam criteria 2003	175	150	Taqman assay	5
Ramezani [35]	2013	Iran	ND	NIH criteria	156	186	RFLP	7
Louwers [24]	2013	Netherlands	ND	Rotterdam criteria 2003	2164	703	GWAS	8
Grigorescu [25]	2013	Romania	ND	ND	177	500	KASPar	5
Yin [23]	2013	China	Chinese	ND	48	96	ND	5

ND: not determined; RFLP: restriction fragment length polymorphism analysis; SSCP: sensitive single strand conformation polymorphism analysis; LDR: ligation detection reaction method; GWAS: genome-wide association study; KASPar: fluorescence-based competitive allele-specific PCR.

**Table 2:** Chromosome position and OR/RR of *INSR* SNPs detected in former studies.

Study	SNP	Position	OR/RR (95% CI)	*p* Value
Conway [26]	rs1799817	7125286	ND	ND
	rs1799815	7125508	ND	ND
	rs1799816	7125507	ND	ND
Talbot [27]	rs2860178	7170506	ND	ND
	rs2860177	7167940	ND	ND
	rs2059806	7166365	ND	ND
	rs2229429	7166377	ND	ND
	rs41509747	7150480	ND	ND
	rs2229431	7141764	ND	ND
	rs41339753	7128812	ND	ND
	rs1799815	7125508	ND	ND
	rs1799817	7125286	ND	ND
Siegel [12]	rs1799817 *	7125286	RR: 2.1 in lean	0.03 in lean, 0.32 in obese
Chen [28]	rs1799817 *	7125286	ND	<0.01 in total and lean, >0.05 in obese
Jin [20]	ND	7125522 *	ND	<0.01
Lee [29]	ND	7184410	ND	ND
	rs6510959	7184227	1.39 (0.77–2.50)	0.2726
	rs2303672	7168394	0.82 (0.42–1.62)	0.5707
	rs2059806	7166365	1.31 (0.86–2.00)	0.2134
	rs2252673	7150407	0.90 (0.61–1.31)	0.5688
	rs2860175	7132070	1.46 (0.82–2.62)	0.2010
	rs1799817	7125286	0.83 (0.56–1.23)	0.3541
	ND	7125064	ND	ND
	rs78312382 *	7117415	0.55 (0.32–0.96)	0.0360
Mukherjee [30]	rs1799817 *	7125286	ND	0.181 in total, 0.004 in lean
Unsal [13]	rs1799817	7125286	ND	0.437
	rs2229434	7142910	ND	1
	rs2229430	7142832	ND	1
	rs16994210	7142988	ND	>0.05
	rs35045353	7142927	ND	>0.05
	rs2162771	7142869	ND	>0.05
	rs1541806	7142844	ND	>0.05
	rs13306451	7142813	ND	>0.05
Hanzu [21]	rs2245648	7163219	ND	>0.05
	rs2962	7163054	ND	>0.05
	rs2245649 *	7163203	ND	0.0086
	rs2963 *	7163143	2.99 (1.41–6.32)	0.0025
	rs2245655 *	7163129	ND	0.0048
Goodarzi [22]	rs12459488	7206062	1.11 (0.92–1.34)	0.3
	rs2971499	7214271	1.05 (0.86–1.28)	0.067
	rs2252673 *	7150407	1.32 (1.08–1.60)	0.006
	rs10401628	7126207	1.00 (0.80–1.25)	0.99
	rs6510949 *	7134391	ND	0.028
	rs919275	7261430	ND	0.089
	rs2042902	7204459	ND	0.099
	rs4804404	7218371	ND	0.101
	rs7254921	7278441	ND	0.111
	rs7258382	7262558	ND	0.164
	rs1549616	7132559	ND	0.166
	rs7248939	7268427	ND	0.190
	rs10408374	7127283	ND	0.249
	rs8103483	7145363	ND	0.300
	rs4804428	7235280	ND	0.360
	rs3745550	7115562	ND	0.372
	rs8112883	7179309	ND	0.404
	rs10426094	7205229	ND	0.420
	rs8110116	7143700	ND	0.511
	rs2860172	7127364	ND	0.521
	rs7245562	7218124	ND	0.522
	rs890862	7233593	ND	0.536
	rs10500204	7182952	ND	0.544
	rs11668751	7251831	ND	0.586
	rs3786680	7183540	ND	0.642
	rs11667110	7136598	ND	0.644
	rs6510960	7203721	ND	0.647
	rs17254521	7238684	ND	0.689
	rs4804195	7254933	ND	0.947
	rs8111710	7292572	ND	0.962
	rs12979424	7273481	ND	0.981
	rs10408844	7244873	ND	0.992
	rs11880337	7296441	ND	ND
	rs6510975	7266867	ND	ND
	rs2860183	7189364	ND	ND
Chen [15]	rs1864193	7114202	0.87	0.164
	rs11667110	7136598	0.92	0.343
	rs16990074	7137340	1.14	0.494
	rs4804304	7140514	1.18	0.387
	rs2229431	7141764	1.03	0.832
	rs2229430	7142832	1.09	0.672
	rs2229434	7142910	1.05	0.814
	rs2962	7163054	1.27	0.35
	rs2059807 *	7166098	1.34	3 × 10^−4^
	rs3815902 *	7166127	1.32	0.002
	rs3786681 *	7168922	1.34	9 × 10^−4^
	rs6510956 *	7169265	1.32	0.002
	rs16994298	7170871	1.26	0.349
	rs8109559	7171618	1.24	0.159
	rs16994314	7176963	1.26	0.354
	rs8108622 *	7182742	1.5	7 × 10^−5^
	rs10500204 *	7182952	1.47	1 × 10^−4^
	rs3786680	7183540	1.21	0.141
	rs891088 *	7184751	1.38	4 × 10^−4^
	rs17253937 *	7184790	1.43	0.001
	rs7245757	7187617	0.94	0.476
	rs1035939	7188968	0.93	0.337
	rs4804368	7190279	0.96	0.602
	rs7254358	7199330	0.91	0.204
	rs1035942	7199792	0.93	0.354
	rs8103883	7203411	1.24	0.075
	rs2042901	7204383	0.91	0.241
	rs10426094 *	7205229	1.34	0.033
	rs12459488	7206062	0.9	0.162
	rs3745546 *	7211805	1.25	0.036
	rs3745545	7211830	1.07	0.584
	rs7245562	7218124	0.93	0.364
	rs7508679	7222821	0.99	0.879
	rs4804415	7223808	1.05	0.636
	rs4804416	7223837	0.97	0.677
	rs7248104	7224420	0.97	0.643
	rs4804424	7229666	1.06	0.553
	rs10416429	7230427	1.03	0.758
	rs890862	7233593	1.05	0.637
	rs10424224	7240470	1.03	0.77
	rs10404318	7247616	0.99	0.944
	rs919275	7261430	1.09	0.283
	rs8101064	7293108	1.15	0.273
Cirilo [31]	rs1799817	7125286	ND	>0.05
Ranjzad [32]	rs2059806	7166365	ND	0.519
	rs1799817	7125286	ND	0.630
Shi [16]	rs2059807 *	7166098	1.14	1.09 × 10^−8^
Kashima [33]	rs1799817 *	7125286	ND	0.308 in total, 0.037 in lean, 0.644 in obese
Skrgatic [34]	rs1799817	7125286	ND	0.631
Ramezani [35]	rs1799817	7125286	ND	>0.05
	rs2059806	7166365	ND	>0.05
Louwers [24]	rs2059807	7166098	0.93	0.27
Grigorescu [25]	rs2059807 *	7166098	3.1 (1.3–6.8) in non-insulin resistant	>0.05 in total, <0.006 in non-insulin resistant
Yin [23]	ND	7125177 *	ND	0.043
	ND	7117386 *	ND	0.055 in total, 0.042 in non-fat
	ND	7117367 *	ND	0.055 in total, 0.042 in non-fat

ND: not determined; *: SNPs that distributed differentially between PCOS and control.

As shown in Table 1, three diagnosis criteria of PCOS were used in different studies. NIH criteria include hyperandrogenism and anovulation. The most widely used Rotterdam criteria require PCOS to be diagnosed by at least two of the following three items: oligo-anovulation, hyperandrogensim and PCO. Some studies all three items to be satisfied for the diagnosis of PCO. For rs1799817, five studies adopted Rotterdam criteria, four adopted the criteria including three items, two adopted NIH criteria, and one failed to document the criteria applied. For rs2059806, two studies adopted NIH criteria, one adopted Rotterdam criteria, and one adopted the three-item criteria. For rs2059807, three studies adopted Rotterdam criteria and one failed to document the criteria applied. BMI was not comparable between controls and PCOS cases in the majority of the studies.

### 2.2. Quality of the Included Studies

As shown in Table 1, the average score of Newcastle-Ottawa quality assessment scale (NOS) was 6.75 (range from 5 to 8). Two items, including the representativeness of the cases and the selection of controls, were the major sources of biases (80%).

### 2.3. Meta-Analysis Results

**Figure 3:**
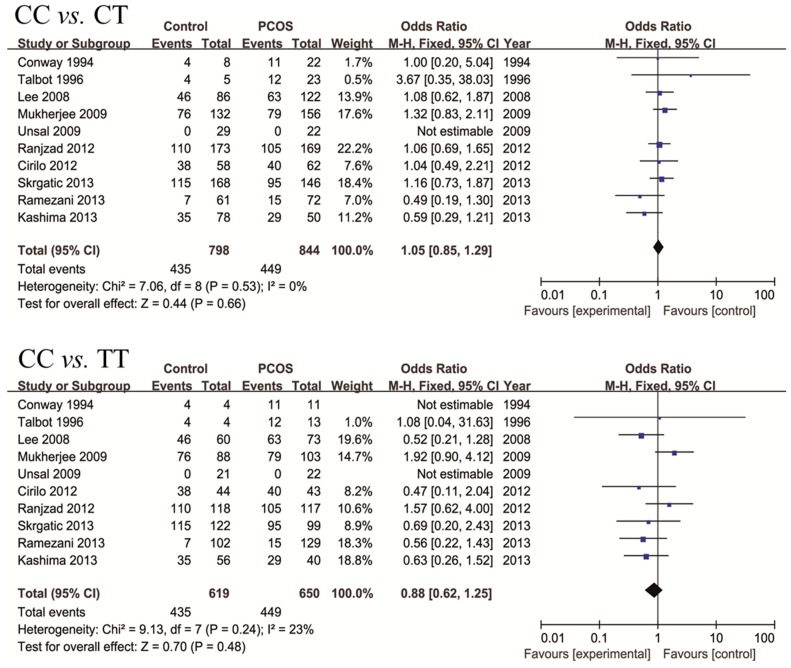

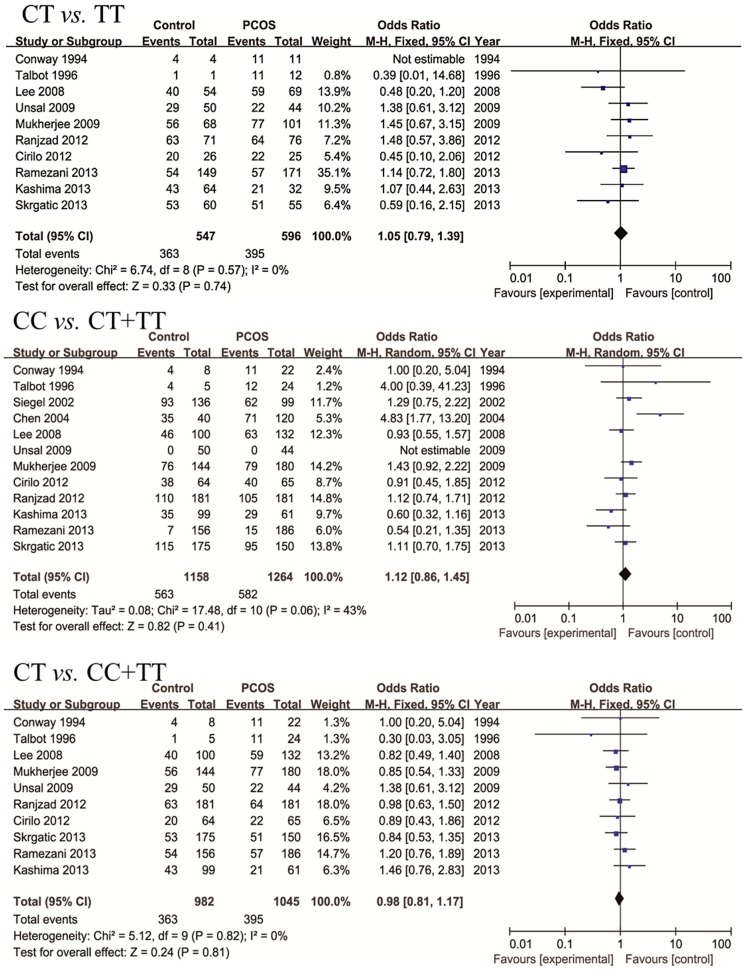

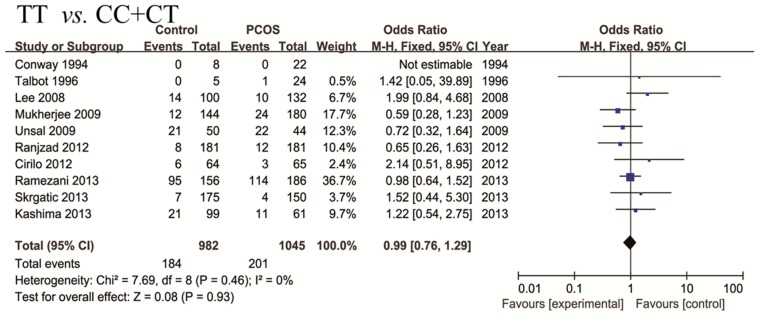
Forest plots of meta-analyses on association between rs1799817 frequency and PCOS risk.

For rs1799817 (Table 3), a total of 12 studies were collected, including 1158 controls and 1264 PCOS cases. Except two studies in which Hardy-Weinberg Equilibrium (HWE) test could not be conducted due to a lack of information and one study that deviated from HWE [13], genotype distribution in the controls was consistent with HWE. In order to explore the potential correlation between genotypes of rs1799817 and PCOS, we compared every genotype (Figure 3). For CC* vs.* CT, CC* vs.* TT, CT* vs.* TT, CT* vs.* CC + TT, and TT* vs.* CC + CT, no significant association was found, with mild between-study heterogeneity. For CC* vs.* CT + TT, a random effect model was selected due to severe between-study heterogeneity (*p* = 0.06), and no significant association was found (*p* = 0.41).

**Figure 4:**
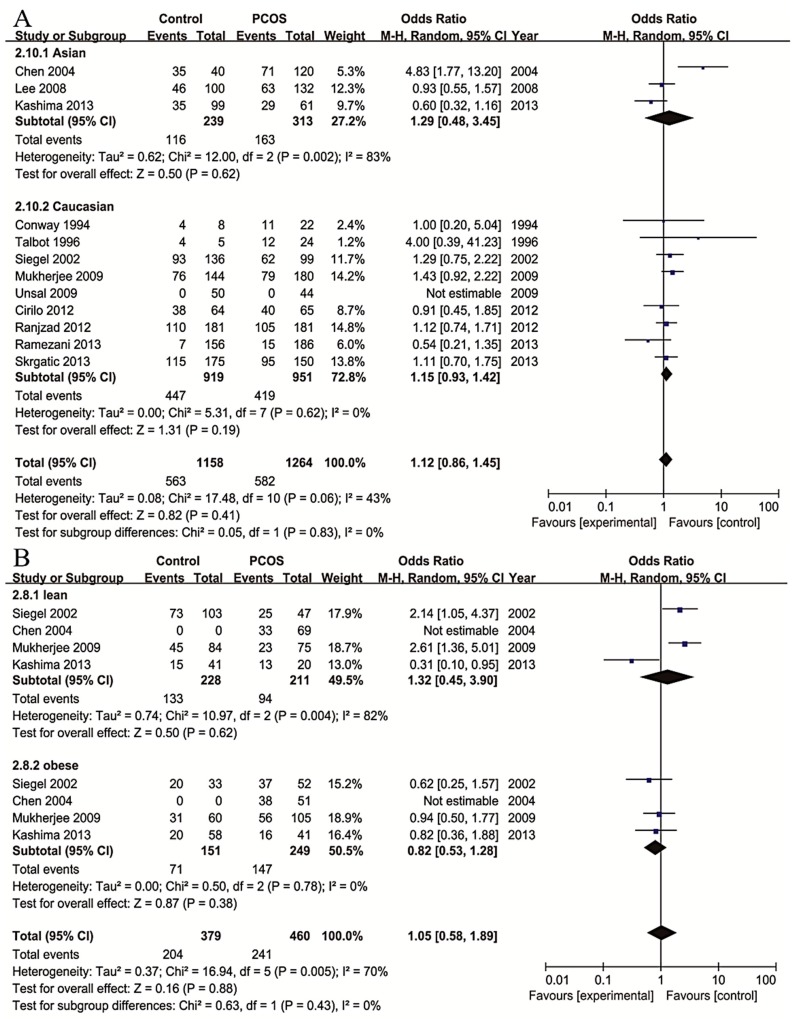

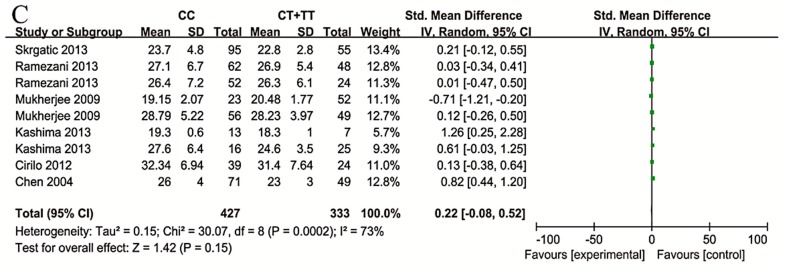
Forest plots of association between genotype frequencies of SNP rs1799817 and PCOS risk. (**A**) Stratified by ethnicity; (**B**) stratified by BMI; (**C**) pooled standardized mean differences of BMI in PCOS patients with different genotypes.

To explore the origin of between-study heterogeneity, subgroup analyses with stratification by ethnicity and weight were carried out (Figure 4). The heterogeneity in the studies from Caucasian population was mild (*p* = 0.62) while the heterogeneity from Asian population was still severe (*p* = 0.002). Both group indicated no significant association (*p* = 0.19 and *p* = 0.62, respectively). Subgroup analyses stratified by weight showed that the heterogeneity in obese population was mild (*p* = 0.78) while in lean population was still severe (*p* = 0.004). The random effect model was selected, and no significant correlation was found (*p* = 0.38 and *p* = 0.62, respectively). Only three studies carried sufficient data to be analyzed in the weight subgroups, and the data was limited to CC* vs.* CT + TT comparison. No correlation was found between rs1799817 and PCOS for CC* vs.* CT + TT, in both lean and obese subgroups. Further analyses indicated no significant difference of BMI between patients with different genotypes (*p* = 0.15). In addition, considering the divergence of diagnostic criteria, we performed the subgroup analysis according to the three diagnostic criteria. Consistent results were obtained from the analyses on groups diagnosed by the same criteria.

**Figure 5:**
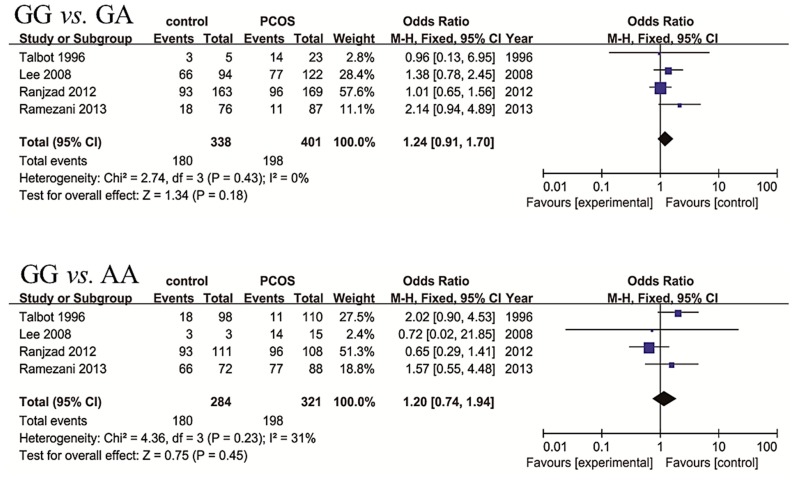

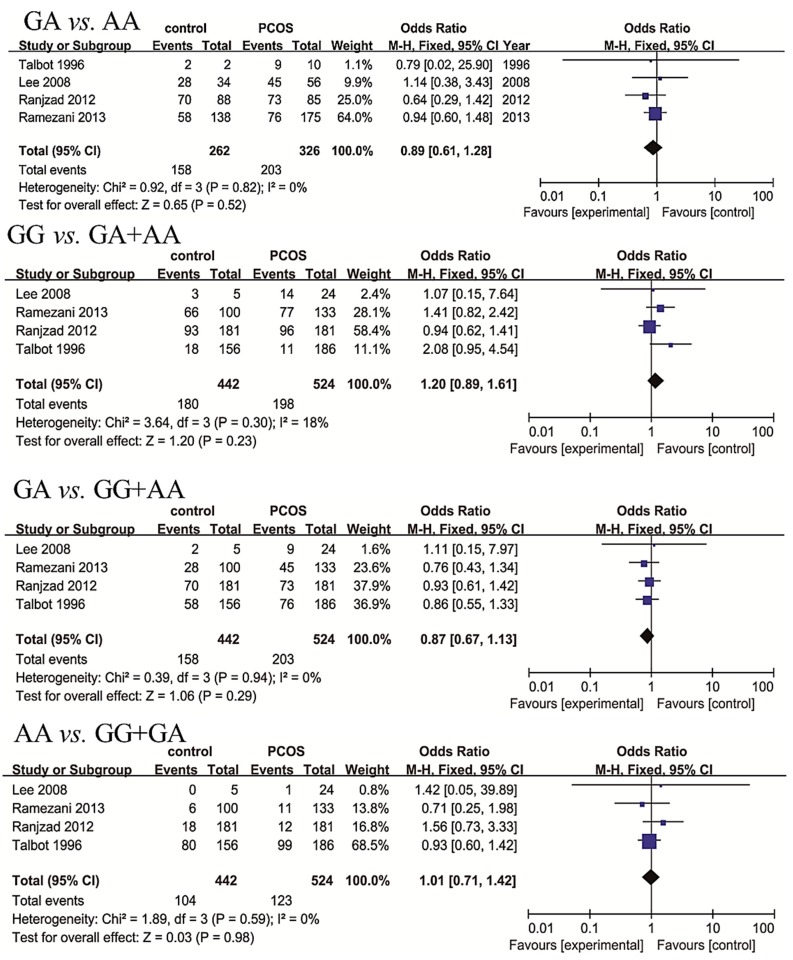
Forest plots of association between genotype frequencies of SNP rs2059806 and PCOS risk.

For rs2059806, four studies including 442 controls and 524 PCOS cases were selected for analyses, (Table 4). The between-study heterogeneity was mild and fixed effect model was applied. No significant association between genotype frequencies of rs2059806 and PCOS was found, including GG* vs.* GA, GG* vs.* AA, GA* vs.* AA, GG* vs.* GA + AA, GA* vs.* GG + AA, and AA* vs.* GG + GA (Figure 5).

Four studies covering 12,830 controls and 11,683 PCOS cases investigated the correlation between rs2059807 genotypes and PCOS. OR of allele C* vs.* allele T was calculated. As shown in Table 5, three studies detected significant correlations while one study found no association. All of these studies were performed in large population, and one of them investigated three cohorts [16]. However, pooled OR could not be calculated because the original information provided was insufficient.

**Table 3:** Distribution of *INSR* rs1799817 genotype and the allele frequencies for controls and PCOS cases.

Study	Year	Country	Age		BMI		Control			PCOS Cases			*p*-Value of HWE
			Control	PCOS	Control	PCOS	Genotypes	Alleles	Cases	Genotypes	Alleles	Cases	
							CC/CT/TT	C/T		CC/CT/TT	C/T		
Conway [26]	1994	UK	ND	ND	23.8 (21.0–24.7)	32.1 (21.3–46.0)	4/4/0	12/4	8	11/11/0	33/11	22	0.641
Talbot [27]	1996	UK	28 (25–33)	24 (18–33)	37.2 (30.5–45.8)	36.6 (27.4–47)	4/1/0	9/1	5	12/11/1	35/13	24	0.970
Siegel [12]	2002	USA	age matched	28 (16–52)	ND	ND	93/43	ND	136	62/37	ND	99	ND
Chen [28]	2004	China	30 ± 4	29 ± 3	23 ± 3	25 ± 4	35/5	ND	40	71/49	ND	120	ND
Lee [29]	2008	Korea	NDs	ND	20.73 ± 2.36	23.22 ± 3.88	46/40/14	132/68	100	63/59/10	185/79	132	0.554
Mukherjee [30]	2009	India	24.94 ± 5.46	24.82 ± 5.26	22.16 ± 4.11	25.01 ± 5.63	76/56/12	208/80	144	79/77/24	235/125	180	0.934
Unsal [13]	2009	Turkey	14.0 ± 3.3	14.5 ± 1.3	20.7 ± 4.2	25.0 ± 5.5	0/29/21	29/71	50	0/22/22	22/66	44	0.015 *
Cirilo [31]	2012	Brazil	32.53 ± 6.91	26.09 ± 6.90	25.61 ± 5.48	31.24 ± 7.15	38/20/6	96/32	64	40/22/3	102/28	65	0.411
Ranjzad [32]	2012	Iran	31.07 ± 5.84	27.13 ± 5.29	25.48 ± 4.21	26.80 ± 6.37	110/63/8	283/79	181	105/64/12	274/88	181	0.964
Kashima [33]	2013	Japan	34.6 ± 5.3	29.5 ± 3.7	20.8 ± 2.6	23.5 ± 5.2	35/43/21	113/85	99	29/21/11	79/43	61	0.528
Skrgatic [34]	2013	Croatia	29.1 ± 4.7	26.7 ± 5.9	22.4 ± 3.3	23.4 ± 4.2	115/53/7	283/67	175	95/51/4	241/59	150	0.960
Ramezani [35]	2013	Iran	30.8 ± 5.6	26.6 ± 5.6	25.5 ± 4.4	26.8 ± 6.4	7/54/95	68/244	156	15/57/114	87/285	186	0.982

* *p* < 0.05; ND: not determine; BMI: body mass index.

**Table 4:** Distribution of the* INSR* rs2059806 genotype and the allele frequencies for controls and PCOS cases.

Study	Year	Country	Age		BMI		Control			PCOS			*p*-Value of HWE
			Control	PCOS	Control	PCOS	Genotypes	Alleles	Cases	Genotypes	Alleles	Cases	
							GG/GA/AA	G/A		GG/GA/AA	G/A		
Talbot [27]	1996	UK	28 (25–33)	24 (18–33)	37.2 (30.5–45.8)	36.6 (27.4–47)	3/2/0	8/2	5	14/9/1	37/11	24	0.855
Lee [29]	2008	Korean	ND	ND	20.73 ± 2.36	23.22 ± 3.88	66/28/6	160/40	100	77/45/11	199/67	133	0.458
Ranjzad [32]	2012	Iran	31.07 ± 5.84	27.13 ± 5.29	25.48 ± 4.21	26.80 ± 6.37	93/70/18	256/106	181	96/73/12	265/97	181	0.673
Ramezani [35]	2013	Iran	30.8 ± 5.6	26.6 ± 5.6	25.5 ± 4.4	26.8 ± 6.4	18/58/80	94/218	156	11/76/99	98/274	186	0.344

ND: not determined.

**Table 5:** Cohort characteristics and odds ratios (ORs) of the *INSR* rs2059807 (C/T).

Study	Year	Country	Title of Cohort	Cases		Age		BMI		OR (95% CI)	*p* Value
				Control	PCOS	Control	PCOS	Control	PCOS		
Chen [15]	2011	China		895	744	30.68 ± 4.68	28.85 ± 3.62	22.68 ± 3.23	24.55 ± 3.99	1.34 (1.16–1.56)	1.16 × 10^−4^
Shi [16]	2012	China	GWAS II	2016	1510	53.95 ± 7.18	28.12 ± 2.75	24.93 ± 3.85	24.59 ± 3.17	1.19 (1.07–1.33)	1.66 × 10^−3^
			REP I	1913	1908	31.29 ± 5.00	28.01 ± 3.01	22.49 ± 2.17	24.23 ± 3.19	1.16 (1.05–1.28)	4.40 × 10^−3^
			REP II	5665	6318	29.52 ± 4.75	27.83 ± 3.11	21.95 ± 2.32	23.72 ± 3.18	1.09 (1.02–1.15)	6.61 × 10^−3^
Louwers [24]	2013	Dutch		2164	703	ND	29.1 ± 5.73	ND	24.8 ± 5.6	0.93	0.27
Grigorescu [25]	2013	Romania		177	500	ND	ND	ND	27.8 ± 0.35	3.1 (1.3-6.8)	<0.006

ND: not determined.

### 2.4. Sensitivity Analysis

Sensitivity analysis was performed by removing one study at one time. For rs1799817, removing any study in the analysis of CC *vs.* CT, CC *vs.* TT, CT *vs.* TT, CC *vs.* CT + TT, CT *vs.* CC + TT, and TT *vs.* CC + CT did not impact the overall results. For rs2059806, when a study from Iran was removed [32], a significant association emerged in the analysis of GG *vs.* GA + AA, with OR 1.57 (1.02–2.42) and *p* = 0.04. No significant association was found from comparison of other genotypes, including GG *vs.* GA, GG *vs.* AA, GA *vs.* AA, GA *vs.* GG + AA, and AA *vs.* GG + GA, in the sensitivity analyses.

### 2.5. Publication Bias

Funnel plots were applied to evaluate the potential publication bias. Visual inspection of the funnel plots indicated no significant asymmetry in analyses for both rs1799817 (Table S1) and rs2059806 (Table S2). This result indicated the absence of severe publication bias.

## 3. Discussion

PCOS is associated with diversified genetic and environmental factors. Multiple candidate genes have been reported to increase the risk PCOS, but often the claims could not be confirmed in validation trials. In the past twenty years, a great number of studies were performed, and a dozen SNPs in different regions of *INSR* gene have been implicated in PCOS, but the results were divergent. For example, for rs2252673, a study by Lee* et al.* [29] indicated no association (*p* = 0.5688) while the study by Goodarzi* et al.* [22] reported a significant association (*p* = 0.006). One reason for the discrepancy could be the limited sample sizes and random errors. In this situation, a meta-analysis pooling together all the available data could effectively reduce the bias and achieve a more reliable conclusion.

Among the *INSR* polymorphisms related to PCOS, rs1799817 SNP is the most thoroughly investigated. INSR contains α and β subunits that are encoded by exons 1–11 and exons 12–22, respectively [36]. Exons 17–22 encode the tyrosine kinase domain, and mutations in this region can cause severe insulin resistance and hyperinsulinemia [37]. SNP rs1799817, located in exon 17, was considered to be involved in insulin resistance and PCOS. In the present study, however, we found no significant correlation between the genotypes of rs1799817 and PCOS. It is noteworthy that Ioannidis *et al*. [14] have performed a meta-analysis on rs1799817 and PCOS in 2010 and same result as current one were obtained in that study. A consistent negative result from previous and current studies support that this genetic variation is unlikely involved in the development of PCOS.

Considering that ethnic group possess diversified genetic and environmental backgrounds, and gene-disease associations may vary across these groups [38], we carried out a further subgroup analysis stratified by ethnicity. No significant difference was found in either Asian or Caucasian populations, indicating that the between-study heterogeneity was not mainly originated from population diversity. Again regarding stratification, previous studies indicated that significant association of genotypes with PCOS was not detected in total population, but observed in lean population. Therefore, we applied a subgroup analysis stratified by body mass index (BMI). Our analyses showed no significant correlation in the lean population. Furthermore, no significant difference was detected in the BMI between CC and CT + TT genotypes in PCOS patients. All together, the results suggested that in both lean and obese groups SNP rs1799817 may contribute little or no to the development of PCOS.

SNP rs2059806 located in the exon 8 of *INSR* gene was investigated in three Caucasian cohorts and one Asian cohort. In the present study, we pooled the results from previous studies and found no evidence that rs2039806 represents a predisposition of PCOS. The sensitivity analysis for GG* vs.* GA + AA showed that, when a study from Iran was removed, an association with PCOS arose with a marginal *p* value. It should be pointed out that the positive results are only based on three studies. Further investigation is needed to clarify the impact of this specific genotype on the risk of PCOS.

While many significant SNPs distribute in different exons and flanking regions of *INSR* gene, we noticed that five SNPs involved in the susceptibility of PCOS concentrate in exon 9 and 5' intron of exon 9, and rs2059807 is one of these SNP located in the “hot spot”. A haplotype constructed with four SNPs in this region was demonstrated to be associated with PCOS [21], indicating that this might be a PCOS susceptibility loci. Unfortunately, no pooled OR could be calculated due to the failure to obtain original data from the groups conducted the studies. Nevertheless, rs2059807 should be considered a candidate risk factor for genetic predisposition of PCOS.

This study has several limitations. First, as mentioned above, since we failed to connect with some authors to collect the original data, the power of the subgroup analysis of BMI was compromised, and the pooled OR of rs2059807 could not be calculated; Second, the ethnicity of some countries could not be clearly defined and we had to classify them as the majority. Similarly, cutoff values for lean and obese were somewhat different among different studies, and we accepted the various definitions by individual studies; Third, original studies used various control groups, including healthy women, infertile women, and elderly women, and various diagnostic criteria of PCOS, making it difficult to control the confounding factors.

In conclusion, our meta-analyses summarized the available data concerning the role of *INSR* polymorphisms for genetic predisposition of PCOS. Our results suggested the absence of significant correlation between rs1799817 or rs2059806 SNPs with the development of PCOS, even if ethnicity and BMI were taken into account. However, rs2059807 could be a promising candidate SNP that might be involved in the development of PCOS. Further investigation is required to clarify the role of rs2029807. Since metabolism abnormality is a major contributing factor for the pathogenesis of PCOS, subgrouped analyses according to BMI and/or insulin resistance status may much enhance the study power.

## 4. Experimental Section

This meta-analysis was developed according to the PRISM statement. A protocol was registered in PROSPERO, with registration number: CRD42014013145.

### 4.1. Literature Retrieval

Two authors independently searched the relevant studies published in all languages via 3 databases, including Pubmed, Embase, and Cochrane CENTRAL no later than 7 August 2014. The discrepancies were resolved by discussion. The following keywords were used: (PCOS OR “polycystic ovary syndrome” OR “polycystic ovarian syndrome”) AND (INSR OR CD220 OR HHF5 OR “insulin receptor”) AND (genotype OR “genetic predisposition” OR SNP OR polymorphism* OR variant* OR “genetic susceptibility” OR genetics OR allele). Additionally, we hand-searched the cited references to obtain more relevant studies.

Studies were included in this analysis if: (1) they evaluated the association between *INSR* SNPs and the risk of PCOS; (2) they were case-control or cohort studies; (3) sufficient data were provided to calculate odds ratio (OR) and 95% confidence interval (CI). Studies were excluded if: (1) they were family-association or case series studies; (2) the studies were from the same research group and the studied populations had overlaps. In this case, only the study with largest sample size was included for analysis and the others were excluded. 

### 4.2. Data Extraction

The following data was extracted from every study by two reviewers independently, and the discrepancies were resolved by discussion: (1) name of the first author; (2) year of publication; (3) country; (4) ethnicity; (5) age; (6) BMI; (7) diagnosis criteria; (8) methods of genotype; (9) SNP detected; (10) OR; (11) *p* values; and (12) numbers of genotypes in cases and controls. We contacted investigators for additional information when extra information was required. When the SNPs were reported with different nomenclatures, we mapped them on the chromosome and unified them into reference SNP ID numbers.

### 4.3. Assessment of Study Quality

The NOS of case control studies was applied to evaluate the quality of studies. Each study was evaluated independently by two authors [39], and they discussed to resolve the disagreement. Three aspects including the selection of population, the comparability of two groups, and the exposure were assessed for eight items, with the highest score of nine.

### 4.4. Statistical Analysis

HWE was tested by the chi-square method. Review Manager 5.2 (Cochrane Collaboration, Oxford, UK) was used for the meta-analysis. The associations of* INSR* SNPs with PCOS were assessed by calculating the ORs and mean difference among the pooled data, and the statistical significance was calculated with Z test. Q test was employed to assess the between-study heterogeneity. A *p* value higher than 0.1 was considered to be of no serious heterogeneity, and a fixed-effect model (FEM) was subsequently applied to calculate the parameters of the data pool [40]. If the heterogeneity is serious, calculation was carried out based on a random-effect model (REM). To define where the severe heterogeneity come from, subgroup analyses on ethnicity and BMI were executed. Additionally, a sensitivity analysis was performed to verify the stability through removing one individual study a time. Publication bias was evaluated by examining the asymmetry of funnel plot. *p* < 0.05 was considered to be statistically significant.

## Supplementary Materials

Supplementary materials can be found at http://www.mdpi.com/1422-0067/16/02/2403/s1.
